# Non-covalently embedded oxytocin in alkanethiol monolayer as Zn^2+^ selective biosensor

**DOI:** 10.1038/s41598-021-85015-w

**Published:** 2021-03-29

**Authors:** Jessica Attia, Sivan Nir, Evgeniy Mervinetsky, Dora Balogh, Agata Gitlin-Domagalska, Israel Alshanski, Meital Reches, Mattan Hurevich, Shlomo Yitzchaik

**Affiliations:** 1 The Institute of Chemistry, The Hebrew University of Jerusalem, Edmond J. Safra Campus, 91904 Jerusalem, Israel; 2 The Harvey M. Krueger Center for Nanoscience and Nanotechnology, The Hebrew University of Jerusalem, Edmond J. Safra Campus, 91904 Jerusalem, Israel; 3 Faculty of Chemistry, Department of Molecular Biochemistry, University of Gdansk, Wita Stwosza 63, 80-308 Gdansk, Poland

**Keywords:** Peptides, Surface assembly, Biosensors

## Abstract

Peptides are commonly used as biosensors for analytes such as metal ions as they have natural binding preferences. In our previous peptide-based impedimetric metal ion biosensors, a monolayer of the peptide was anchored covalently to the electrode. Binding of metal ions resulted in a conformational change of the oxytocin peptide in the monolayer, which was measured using electrochemical impedance spectroscopy. Here, we demonstrate that sensing can be achieved also when the oxytocin is non-covalently integrated into an alkanethiol host monolayer. We show that ion-binding cause morphological changes to the dense host layer, which translates into enhanced impedimetric signals compared to direct covalent assembly strategies. This biosensor proved selective and sensitive for Zn^2+^ ions in the range of nano- to micro-molar concentrations. This strategy offers an approach to utilize peptide flexibility in monitoring their response to the environment while embedded in a hydrophobic monolayer.

## Introduction

Biosensors play a major role in biomedical diagnosis^1^. The well described applications of biosensors contribute to progress in the clinical, environmental and agricultural industries^2^. Biosensors are analytical devices that perform two functions: recognition of an analyte and transduction to a measurable signal^3^. Electrochemical biosensors are commonly used for label-free sensing of bio-recognition events^4^. Electrochemical impedance spectroscopy (EIS) is an electrochemical technique sensitive to changes in the sensory layer’s electronic properties^5,6^. EIS often used to measure a recognition event between a biomolecule and an analyte because, upon binding, the sensory layer undergoes morphological changes that result in alternations of interface properties such as charge, hydrophobicity, capacitance, and surface density. These changes affect the permeation ability of the RedOx agent through the layer, thus, results in detectable impedimetric signal^5,7–9^.

Peptides are often used as recognition entities in biosensors due to their specific affinity towards many moieties, which are crucial for their biological activity^10–12^. Peptides are specifically attractive for EIS since they undergo massive conformational and dipole changes upon analyte binding resulting in measurable signal^12–14^. Their selectivity towards analytes such as proteins^15^, glycans^16,17^, small molecules^18^, and ions^19^ can be controlled by changing the sequence of amino acids. The multi-functionality of the peptide can also enable anchoring to a surface without harming their affinity towards a specific analyte^20,21^. Such decoupling enables impedimetric sensing on differently modified sensing platforms^22^.

Oxytocin (OT) is a neuropeptide with an affinity to both Zn^2+^ and Cu^2+^ ions^23^. Moreover, binding these ions is essential for OT biological activities^24,25^. Zn^2+^ and Cu^2+^ ions bind OT via different mechanisms and do not utilize the same binding sites for the interactions^23,26,27^. Namely, the terminal amine is essential for activation of OT-Cu^2+^ and initiates binding cascade, which proceeds with deprotonation of backbone amides^23,28,29^. Chelation of Zn^2+^ in OT proceeds via interactions with amides carbonyls^23,28^. Each of these metal ions induces distinctive conformational, electronic, and hydrophobicity changes to the OT complex^24,30^. Therefore, OT selectivity and sensitivity can be tuned for biosensing applications of Zn^2+^ and Cu^2+^^20,31,32^.

In our previous studies, we described several types of electrochemical OT-based biosensors for Zn^2+^ and Cu^2+^ ions. In these models, oxytocin was either anchored to a gold surface through a native disulfide bond^20^ or attached to a glassy carbon electrode using a silane-coupling strategy^33^. We showed that by coupling OT through terminal amine to lipoic acid, the affinity towards Cu^2+^ was blocked and the selectivity towards Zn^2+^ was improved^32^. This sensor proved highly selective but suffered from low surface charge transfer resistance (R_CT_) alternation, which can be explained by the low impedimetric signal-to-noise ratio (SNR) in the system. Low surface coverage is usually associated with peptides assembly since they are flexible, in constant conformational equilibrium/shift and experience repulsion from neighbouring peptides^34,35^. Contrary, long alkyl thiols pack to a very dense monolayer on gold surfaces and can act as a host layer for hydrophobic moieties^36,37^. We hypothesize that a recognition of analyte by a peptides embedded within a dense host layer may lead to significant changes in the properties of entire sensory layer and result in enhancement of the impedimetric signal.

In this study we present a biosensing platform in which OT functionalized with a dodecanoic acid (Dd) was embedded in a host alkane thiol monolayer by non-covalent interactions. OT functionalization leads to both selectivity toward Zn^2+^ ions *due* to terminal amine blocking and also to interlayer affinity by introducing alkane chain. The presented strategy extrapolates the formed hydrophobic surface of OT after Zn^2+^ chelation to change peptide interactions with the hexadecane thiol (HDT) layer and thus produce a measurable and concentration-dependent impedimetric signal. We envisioned that the effect of the peptide-metal ion chelation on the morphological changes in the sensory layer might lead to amplification of the impedimetric R_CT_ parameters and hence could be applied for improving electrochemical biosensing. These bioactive intercalated monolayers were characterized by atomic force microscopy (AFM), variable angle spectroscopic ellipsometry (VASE), contact angle (CA), X-ray photoelectron spectroscopy (XPS) to study the interplay between surface morphology and the impedance.

## Results and discussion

### Platform design

**Figure 1:**
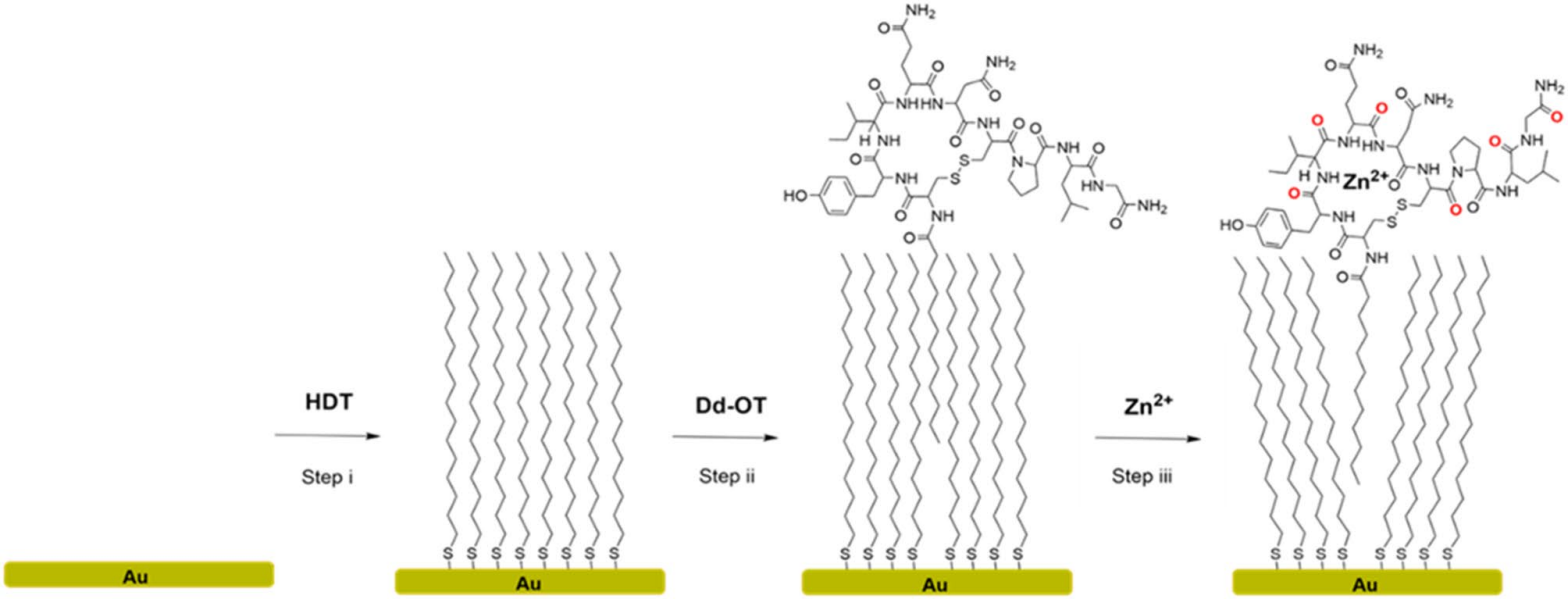
The fabrication steps of the sensing platform. *Step i*, self-assembly of HDT monolayer. *Step ii*, intercalation of Dd-OT molecule in the HDT SAM. *Step iii*, OT-Zn^2+^ complexation.

OT was synthesized by solid-phase peptide synthesis in which dodecanoic-acid was coupled to the OT-terminal amine (see SI, Figure S1). The obtained Dodecanoic-Oxytocin (Dd-OT) designed to facilitate embodiment to the intercalation to the HDT self-assembled monolayer (SAM) on gold substrate by van der Waals (vdW) interactions between alkyl groups (Fig. 1).

### Characterization of the system

**Figure 2:**
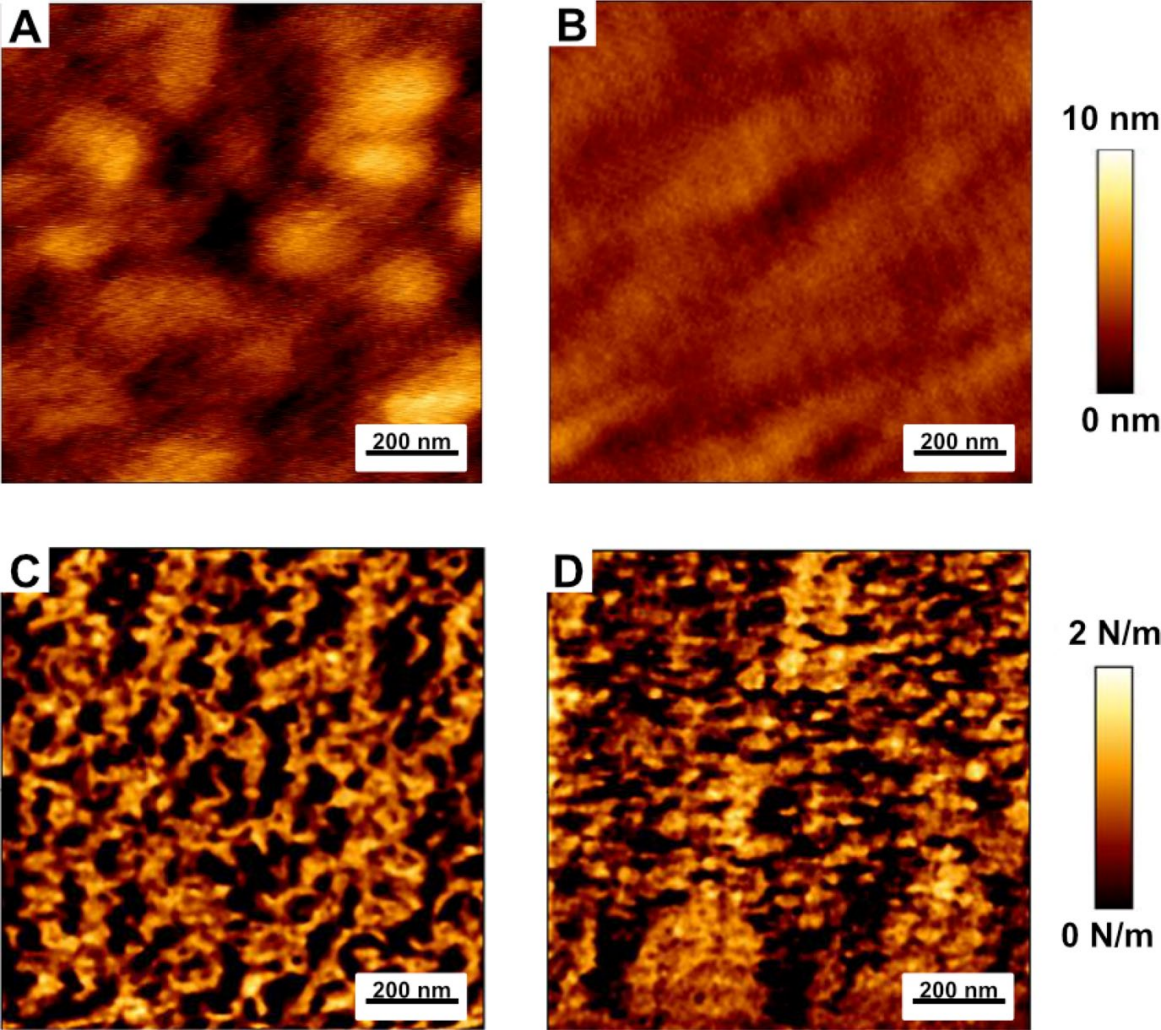
Zinc ion binding influence on the morphology and the stiffens of the layer: AFM topography analysis of (**A**) HDT-Dd-OT (Ra = 1.05 ± 0.09 nm) and (**B**) HDT-Dd-OT-Zn^2+^ (Ra = 0.52 ± 0.05 nm) and Slope of force-distance images of (**C**) HDT-Dd-OT and (**D**) HDT-Dd-OT-Zn^2+^.

Au-evaporated layer on Si wafers was modified with HDT SAM following incubation with Dd-OT and further exposure to Zn^2+^ (Fig. 1). These modified wafers were characterized by VASE, AFM, CA, and XPS. The HDT formed homogenous typical and well described^38,39^. SAM with measured thickness of 1.9 ± 0.1 nm, CA of 104° ± 2°, and roughness of 1.8 ± 0.1 nm (Figure S3). These substrates were incubated with Dd-OT and analyzed by XPS. The analysis (Figure S7) confirmed the presence of amide bonds related to Dd-OT at BE of 400.2 eV (related to N1s 1/2)^40^ and carbonyl groups (Figure S8) by the peak of 288.5 eV (related to C1s/8). The embedding of the layer with Dd-OT resulted in a total layer thickness of 3.4 ± 0.2 nm and roughness of 1 ± 0.1 nm (Fig. 2A). The measured increase in thickness and the decrease of roughness indicate that embedding Dd-OT changes the morphological properties of the host HDT SAM on Au substrate. However, the CA with a value of 103° ± 1° indicated that the change in hydrophobicity *due* to the assembly of the peptide on the host layer is insignificant.

Exposure of the sensory layer to Zn^2+^, which is the analyte of the Dd-OT recognition element, resulted in the appearance of an XPS peak (Figure S9) with BE of 1022.3 eV (related to Zn2p 3/2), which is shifted relative to free Zn^2+^, that indicates the presence of the chelated Zn^2+^^20,41^.  AFM analysis indicated a roughness decrease to 0.5 ± 0.1 nm (Fig. 2B), while CA of 94° ± 1° related to a decrease in hydrophobicity. No significant thickness changes were observed by VASE analyses upon exposure for Zn^2+^. The decrease in hydrophobicity may indicate that a more hydrophilic layer containing the charged Dd-OT-Zn^2+^ was formed. The roughness alternation, which was measured by AFM (Fig. 2A,B), indicates the morphological changes in the layer *due to* Zn^2+^ binding^20,32,42^. The fact that VASE measurements did not show any changes in the thickness of the layer indicates that there are no significant changes in the positioning of the peptide in relation to the host layer. AFM with a quantitative mode of operation (QI) was performed to calculate stiffness values and Young’s modulus of Dd-OT modified substrates before and after exposure to Zn^2+^ (Fig. 2C,D and Figures S4–S6). The exposure to Zn^2+^ caused an increase in Young’s modulus from 2 ± 1 GPa to 4.8 ± 0.5 GPa, indicating that Zn^2+^ results in morphological changes of the monolayer^43^. The changes in surface morphology and other presented parameters caused by the binding of Zn^2+^ on the biosensing platform, can be further applied in impedimetric biosensing, which is a sensitive technique to exploit changes in layer morphology.

### Impedimetric analysis of HDT SAM and Dd-OT intercalation

**Figure 3:**
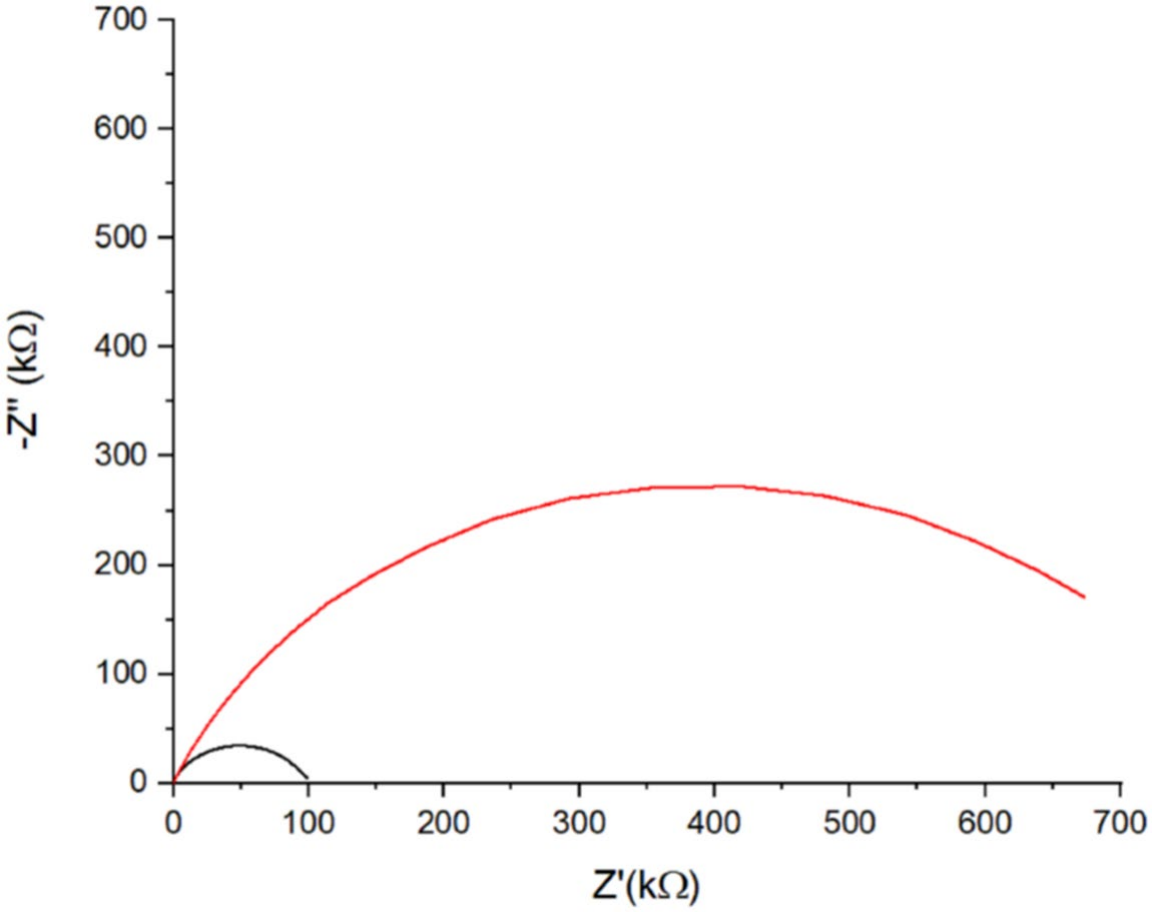
Nyquist plot of HDT-modified electrode before (black curve) and after (red curve) Dd-OT intercalation.

Au working electrodes were modified with HDT SAM by the presented protocol and analyzed by EIS measured in a solution containing [Fe(CN)_6_]^4−^/[Fe(CN)_6_]^3−^. This modification resulted in an increase of R_CT_ from 0.2 kΩ for bare Au electrode (Figure S2) to 89 kΩ for HDT SAM (Fig. 3) indicating the formation of a dense layer^44^. After the immersion of the HDT-modified electrode in Dd-OT solution, the R_CT_ increased to 697 kΩ, which is much higher than the value observed for OT covalently attached to the surface^20,32^. This large increase in the R_CT_ values can be attributed to changes in layer morphology such as density, stiffness, rigidity, etc. caused by intercalation of HDT with Dd-OT^2,4–6,9^.

### Dose–response of Dd-OT sensor to Cu^2+^ and Zn^2+^ ions

**Figure 4:**
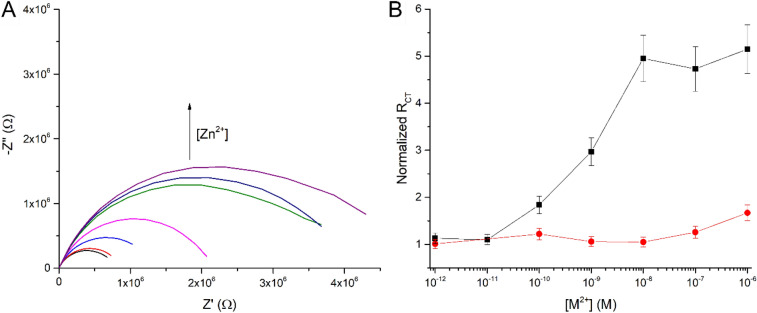
Nyquist plots for (**A**) different Zn^2+^ ion concentrations and (**B**) dose–response of Dd-OT sensor to Zn^2+^ (black curve) and Cu^2+^ (red curve) ions. The presented averaged values are based on measurements from three different electrodes (Figure S10).

HDT-Dd-OT sensor was exposed to Zn^2+^ solutions with increasing concentrations from 10^–12^ M to 10^–6^ M (Fig. 4). The exposure to Zn^2+^ concentrations lower than 10^–10^ M had no considerable effect on the R_CT_ values, which is below the limit of detection of the presented sensor. At concentrations ranging from 10^–10^ M to 10^–8^ M, the impedimetric response gradually increased and eventually reached a value five times higher than the measured R_CT_ values prior to Zn^2+^ exposure (Fig. 4B). At higher concentrations (above 10^–8^), the sensor reached its saturation and the signal stabilized. The exposure of the intercalated OT layer to 10^–8^ M of Zn^2+^ (HDT-Dd-OT-Zn^2+^) resulted in a signal enhancement that reached an R_CT_ value which is five times higher than that of the surface prior to incubation with the ion (HDT-Dd-OT). This signal enhancement surpasses the response following exposure to Zn^2+^ of chemisorbed or covalently bound OT surface layers, which only reached an increase of up to 1.35 in R_CT_ values over the same range of concentration^32^. We assume that the signal enhancement observed is because the OT complexation with Zn^2+^ influences not only the peptide conformation itself but also modifies the morphology and the net charge of the entire sensory layer. This directly influences the permeation of the redox species through the intercalated layer. To verify the selectivity of the sensor, dose–response analysis for Cu^2+^ ions was performed. No significant impedimetric response was observed in the relative concentration range (10^–12^ M to 10^–6^ M; see Fig. 4, Figure S10). This demonstrates that the dodecyl functionalization of OT by amidation of the terminal amine blocks its ability to complex Cu^2+^, as previously reported^32^.

## Conclusions

In this work, we designed a new Zn^2+^ ion-selective receptor based on an alkyl-amidated OT embedded in a host alkanethiol monolayer by vdW interactions. This intercalated peptide monolayer induces enhanced impedimetric response upon ion-binding. Its response to Zn^2+^ induced conformational changes that evoked morphological changes to the entire monolayer. These alterations were probed by impedimetric measurements and yielded a selective sensor for Zn^2+^ ions in the range of 0.1 nm up to 10 nm. By this, the monolayer serves as a host for the Dd-OT sensing peptide, which enhances the R_CT_ multiplication for the biorecognition event. This approach is fundamentally different from sensors assembled by direct covalently bound OT monolayers. This non-covalent assembly concept can be further extended to other bio-receptors incorporated in natural and artificial membranes.

## Materials and methods

### Surface modifications

OT was synthesized using solid-phase peptide synthesis (SPPS) and modified by coupling dodecanoic-acid (Dd) to the N-terminal using the same method (see SI). Au layer (100 nm) was evaporated on top of a Cr layer (10 nm), which was evaporated on the substrate of n-type Si wafer ($\left\langle {100} \right\rangle$). The bare gold surfaces were washed with absolute ethanol, before and after 20 min cleaning using Ultra Violet Ozone Cleaning Systems (UVOCS Inc). HDT (CH_3_–(CH_2_)_15_–SH, Sigma-Aldrich) was freshly distilled under vacuum before use. HDT adsorption was performed by immersing bare gold surfaces in HDT solution (10 mM in absolute ethanol) for 18 h at 22 °C. After incubation, the surfaces were washed with absolute ethanol and dried under dry N_2_. Dd-OT was dissolved in ammonium acetate (AA) buffer (50 Mm, pH = 7) (Sigma-Aldrich). Then, HDT-modified surfaces were immersed in the Dd-OT solution (10 µM) overnight at 22 °C to allow the intercalation of Dd-OT with the HDT SAM. Afterward, the surfaces were washed by immersion in AA buffer for 10 min and dried under dry N_2_. Complex formation of Dd-OT with either Zn^2+^ or Cu^2+^ ions was obtained by incubating the modified surfaces in 10 µM Zn^2+^/Cu^2+^ in AA buffer for 10 min.

### Ellipsometry

Thickness measurements were performed by variable angle spectroscopic ellipsometry (VASE) measurements with VB-400 ellipsometer (Woollam, Lincoln, NE, USA) at the Brewster angle of 75^**°**^.

### Atomic force microscopy (AFM)

Measurements were carried out by NanoWizard3 (JPK Instruments, Berlin, Germany) in fresh AA buffer (pH = 7) at 298 K, using Aspire CT-130 (Team Nanotech, Villingen-Schwenningen, Germany) cantilevers with a spring constant of 6 N/m under QI mode—force curve-based imaging. To assure homogeneity of the surface, images were taken at different regions of the same surface and at a resolution of 500 × 500 pixels, an area of 2 × 2 μm and at a pixel rate of 0.8 Hz. Young’s modulus and stiffness values were calculated by JPK Instrument data analysis software using Derjaguin-Muller-Toporov (DMT) model and the force curve slope.

### Electrochemical impedance spectroscopy (EIS)

Electrochemical analyses were conducted by Metrohm-Autolab PGSTAT-302N digital potentiostat (EcoChemie BV, Utrecht, Netherlands) operated by Nova software. The electrochemical cell contained three electrodes: Ag/AgCl/3M KCl as a reference electrode, Pt as a counter electrode, and a polycrystalline disc gold electrodes with a 2 mm diameter (CH instruments) The electrolyte solution was prepared from AA buffer (50 mM, pH = 7) which contained 0.1 M KCl as a supporting electrolyte, and 5.0 mM K_3_[Fe(CN)_6_], 5.0 mM K_4_[Fe(CN)_6_] as RedOx species. The gold electrodes were cleaned by polishing with 0.05 µm alumina suspension on micro-cloth pads (Buehler). Electrode modifications were performed following the precedent described procedures. The frequency range was 0.1 Hz–10 kHz for HDT-modified electrode measurement, and 0.01 Hz–10 kHz for Dd-OT-modified electrode measurement. After dip—coating, the HDT-modified electrode was washed with ethanol absolute and the Dd-OT-modified electrode with AA buffer, until stabilization of the impedance response. Then, dose–response of the sensor was conducted by 10 min incubation of the Dd-OT-modified gold electrode in Zn^2+^/Cu^2+^ solution (1 pM–10 µM at room temperature). After each EIS measurement, the modified electrode was washed and moved to a higher concentration. The equivalent circuit chosen to fit EIS data was R_S_(Q[R_CT_|W]), with R_S_ value for solution's resistance, Q for layer’s capacitance, R_CT_ for charge transfer resistance of the layer, and W for Warburg impedance. The results are presented as normalized R_CT_. The charge transfer resistance was normalized by dividing the R_CT_ value after exposure to heavy metal ions by the R_CT_ value before exposure.

## Supplementary Information

Supplementary Information.

## Data Availability

All data are available upon request.
