# Supplementary material for: Non-covalently embedded oxytocin in alkanethiol monolayer as Zn2+ selective biosensor
Source: Sci Rep. 2021 Mar 29;11:7051. doi: 10.1038/s41598-021-85015-w (PMC8007701; doi:10.1038/s41598-021-85015-w)
Supplement: Supplementary file 1 — Supplementary Information. [file 41598_2021_85015_MOESM1_ESM.docx]

Supplementary Information

**Non-covalently embedded oxytocin in alkanethiol monolayer as Zn^2+^ selective biosensor**

*Jessica Attia,^1,2,4^ Sivan Nir, ^1,2,4^ Evgeniy Mervinetsky,^1,2,4^ Dora Balogh, ^1,2^ Agata Gitlin-Domagalska,^1,3^ Israel Alshanski,^1,2^ Meital Reches,^1,2^* Mattan Hurevich,^1,2^* and Shlomo Yitzchaik ^1,2^**

^1^ The Institute of Chemistry, The Hebrew University of Jerusalem, Edmond J. Safra Campus, Jerusalem 91904, Israel.

^2^ The Harvey M. Krueger Center for Nanoscience and Nanotechnology, The Hebrew University of Jerusalem, Edmond J. Safra Campus, Jerusalem 91904, Israel.

^3^ Faculty of Chemistry, Department of Molecular Biochemistry, University of Gdansk, Wita Stwosza 63, 80-308 Gdansk, Poland.

^4^ These authors contributed equally

Email: [Shlomo.Yitzchaik@mail.huji.ac.il](mailto:Shlomo.Yitzchaik@mail.huji.ac.il)

Email: [Mattan.hurevich@mail.huji.ac.il](mailto:Mattan.hurevich@mail.huji.ac.il)

Email: [Meital.reches@mail.huji.ac.il](mailto:Meital.reches@mail.huji.ac.il)

**Dodecanoic-oxytocin synthesis**

Oxytocin was synthesized by microwave assisted solid-phase peptide synthesis (SPPS) applying Fmoc chemistry using Fmoc-RINK-MBHA resin (loading 0.71 mmol/g).

The peptide chain was elongated in sequential cycles of deprotection and coupling. Deprotection was performed with 20% piperidine in DMF and 5 eq of the protected amino acid derivatives during the first coupling were used, in one of the following mixtures:

Fmoc-AA/ HBTU/ HOBt/ DIPEA, molar ratio 1:1:1:2,

Fmoc-AA/ HATU/ HOAt/ DIPEA, molar ratio 1:1:1:2.

Each coupling and deprotection were followed by Kaiser test *[Kaiser E. et al., Anal Biochem, 1970]* to confirm reaction completion. The coupling was repeated if needed.

After the attachment of *N*-terminal Fmoc-Cys(Trt) and removal of the Fmoc protecting group, dodecanoic acid was introduced. 3 eq of dodecanoic acid and the mixture of HATU/HOAt/ DIPEA, molar ratio 1:1:2 were applied, reactions was performed for 90 min at room temperature. A positive result of the Kaiser test *[Kaiser E. et al., Anal Biochem, 1970]* indicated incomplete coupling, thus reaction was repeated using the same mixture, but 1.5 eq of dodecanoic and was performed overnight.

After completion of the synthesis confirmed by negative Kaiser test, a peptide with attached fatty acid in the *N*-terminus, was cleaved from the resin simultaneously with the side chain deprotection in a one-step procedure. Therefore, the dried resin was suspended in the mixture of TFA/H_2_O/PhOH/TIPS (88:5:5:2, v/v/v/v) and stirred for 3 hours at room temperature *[Pearson D. A., Tetrahedron Lett.,* ***1989****, 30, 2739–2742].* Subsequently, the disulfide bridge was formed using a 0.1 m methanolic iodine solution *[Yang Y., Protein Sci.,* ***1994****, 3, 1267-1275.].*

The crude product was purified by RP-HPLC (Merck-Hitachi, Japan), using a XSELECT CSH 130Prep preparative RP column (C18, 19 × 150 mm, 5 μm). The solvent systems were TDW (0.1% TFA) (A) and ACN (0.1% TFA) (B).

The purity of the synthesized compound was evaluated by RP-HPLC using Zorbax RX-C18 analytical RP column (C18, 4.6 × 150 mm, 5 μm) and was carried out on a Merck-Hitachi HPLC, monitored at 226 nm. The solvent systems were TDW (0.1% TFA) (A) and ACN (0.1% TFA) (B). The mass spectrometry analysis was performed using ESI-MS on LCQ Fleet Ion Trap mass spectrometer (Thermo Scientific).

**Figure S1.** MS of Dd-OT with peaks of 1189.42, 1211.58 corresponding to calculated m/z +H^+^ and m/z +Na^+^ respectively

300

400

500

600

700

800

900

1000

1100

1200

1300

1400

1500

1600

1700

1800

1900

2000

m/z

0

5

10

15

20

25

30

35

40

45

50

55

60

65

70

75

80

85

90

95

100

1189.42 [M + H]^+^

1211.58 [M+ Na]^+^


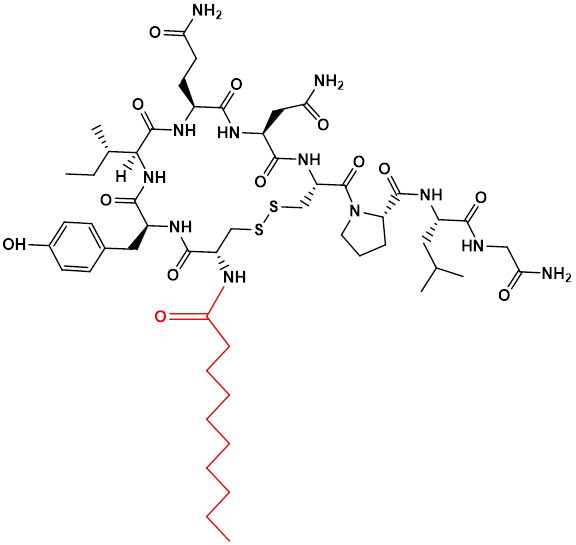


Dodecanoic-Oxytocin

Exact Mass: 1188.6035

Molecular Weight: 1189.5000


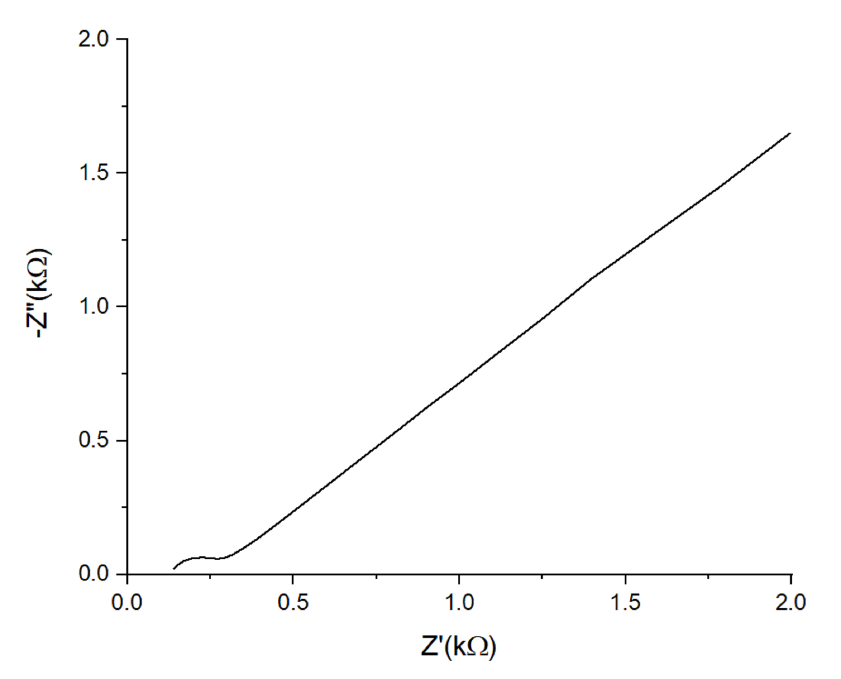


**Figure S2.** Nyquist plot of a bare Au electrode, R_CT_ of [Fe(CN)_6_]^4-^/[Fe(CN)_6_]^3-^ is 200 Ω.

**
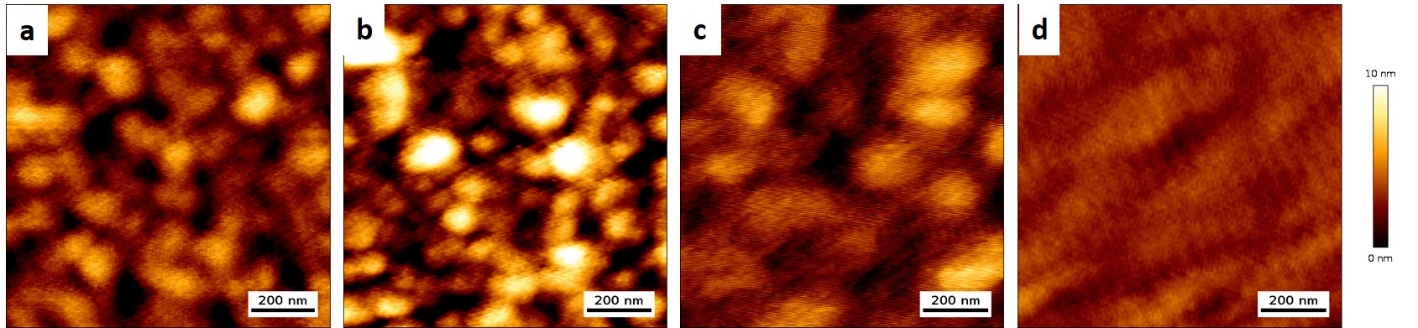
**

**Figure S3.** Topography analysis obtained by tapping mode. (a) Au surface, Ra=1.15±0.05 nm, and Au surfaces modified by: (b) HDT, Ra= 1.8±0.1 nm, (c) HDT-Dd-OT, Ra=1.0±0.1 nm and (d) HDT-Dd-OT after exposure to 10 µM Zn^2+^ solution in AA buffer, Ra=0.52±0.05 nm


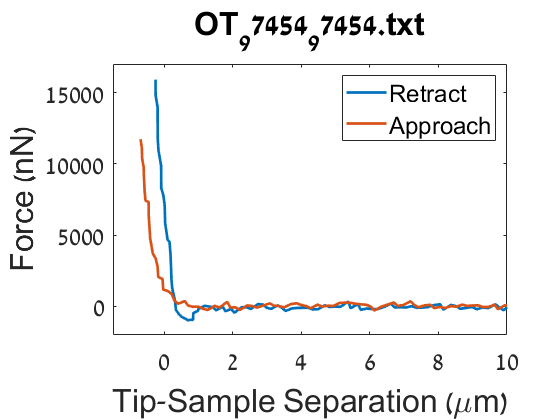

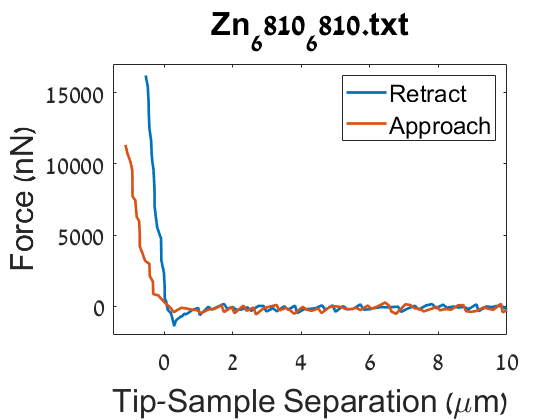


**a**

**b**

**Figure S4.** Force-distance curves obtained by QI mode of Au surfaces modified by: (a) HDT-Dd-OT and (b) HDT-Dd-OT after exposure to 10 µM Zn^2+^ solution in AA buffer.**
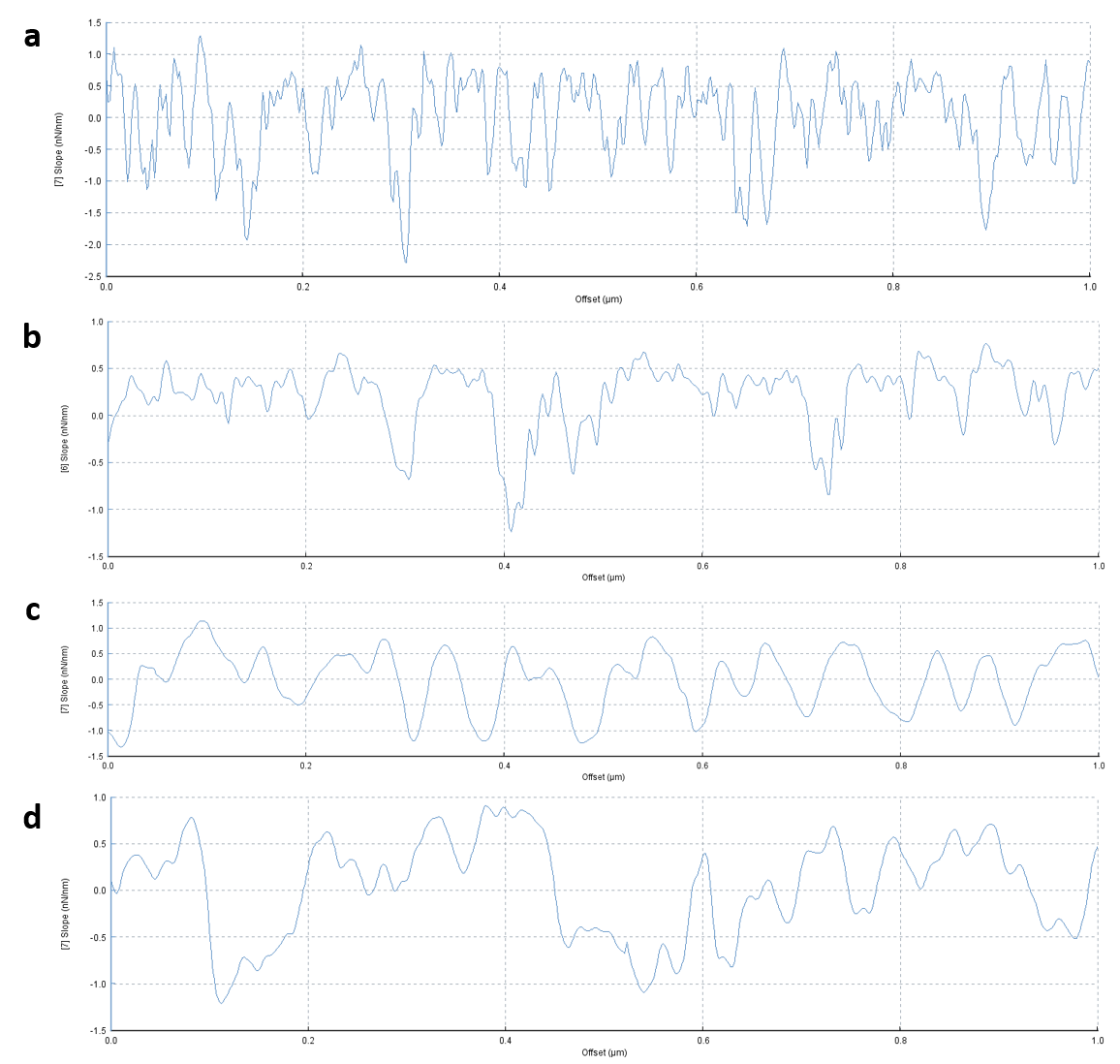

Figure S5.** Cross sections of slope images presenting the variability in stiffness values: (a) Dd-OT and (b) Dd-OT-Zn­^2+^

b

a

**
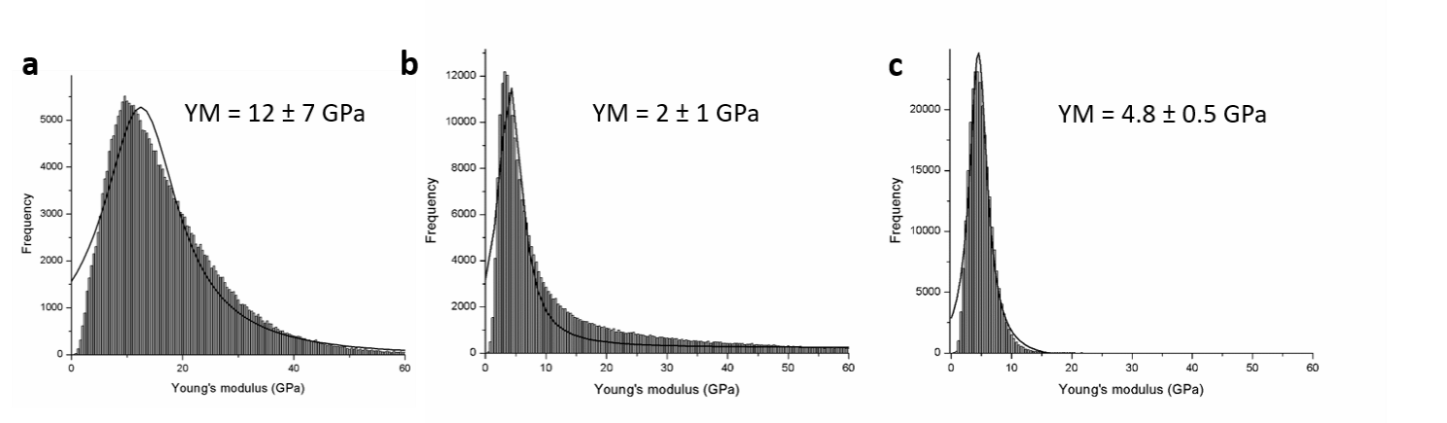
**

b

a

**Figure S6.** Histograms of the distribution of Young’s modulus calculated from each pixel of (a) Dd-OT and (b) Dd-OT-Zn^2+^ surfaces.

- HDT

- HDT-Dd-OT

**Figure S7.** N 1s integration by XPS analysis of HDT (green curve) and HDT-Dd-OT (red curve) layers

- HDT

-HDT-Dd-OT

_

C

1

s

/

8

_

C

1

s

(

CH

)

_

C

1

s

(

C

-

O

)

_

Pos

.

_

285

_

286

.

769

_

Area

_

1068

.

8

_

24

.

9

_

%

Area

_

97

.

73

_

2

.

27

1

s

(

CH

)

_

C

1

s

(

C

-

O

)

_

C

1

s

(

C

=

O

)

_

Pos

.

_

285

_

286

.

168

_

288

.

526

_

.

_

464

.

5

_

216

.

8

_

%

Area

_

57

.

33

_

29

.

09

_

13

.

58

x 10

1

60

70

80

90

100

110

120

130

140

150

CPS

294

292

290

288

286

284

282

280

278

276

Binding Energy (eV)

**Figure S8.** C 1s integration by XPS analysis of HDT layer (green curve) and HDT-Dd-OT layer (red cureve)



 **Figure S9.** Zn 2p integration (red fit) by XPS analysis of HDT-Dd-OT layer (black curve) and HDT-Dd-OT layer after exposure to 10 µM Zn^2+^ solution (grey curve)


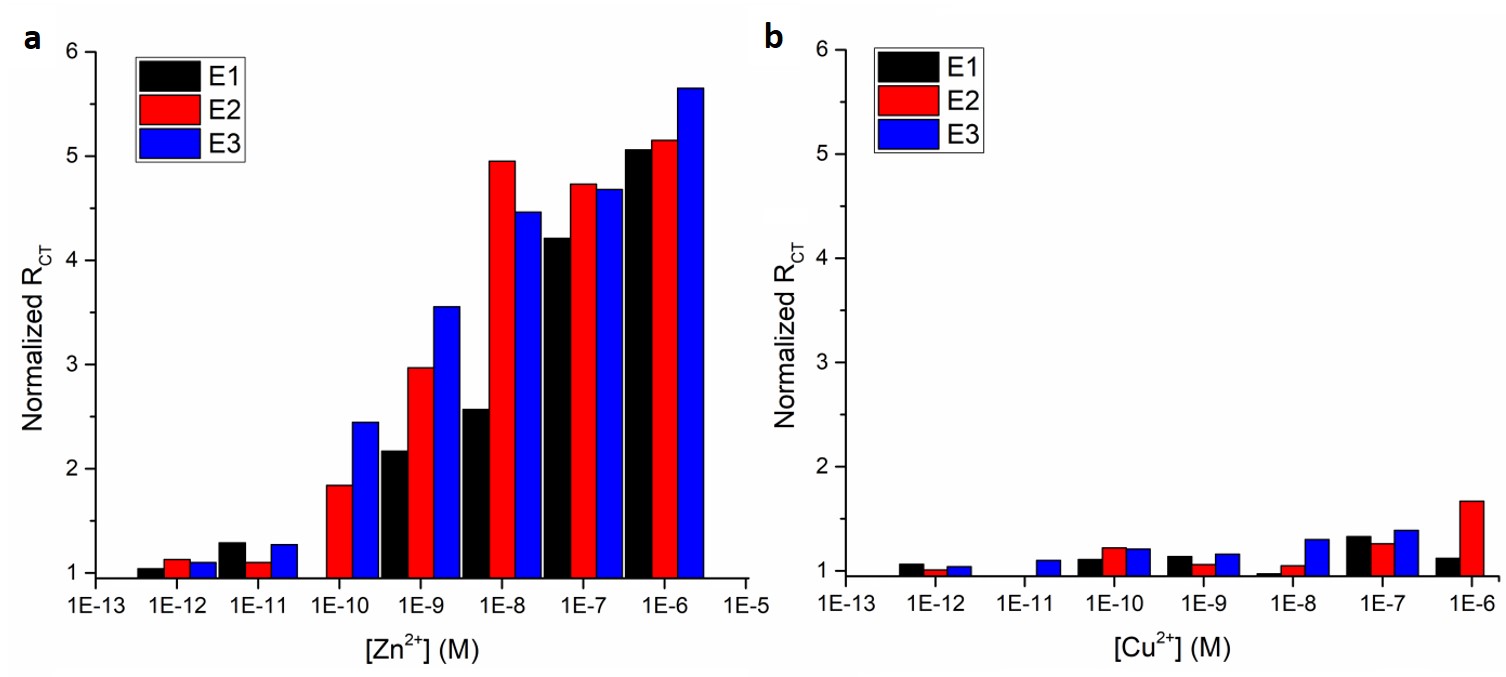


**Figure S10.** Response of 3 different electrodes to increasing concentration of (a) Zn^2+^ and (b) Cu^2+^
